# Wheezing in low birth weight infants

**DOI:** 10.1186/1939-4551-8-S1-A163

**Published:** 2015-04-08

**Authors:** Gustavo Falbo Wandalsen, Herberto Jose Chong Neto, Carolina Aranda, Ana Caroline Dela Bianca, Javier Mallol, Dirceu Sole, EISL Group Brazil

**Affiliations:** 1 Federal University of Sao Paulo, Brazil; 2 Universidade Federal Do Paraná, Brazil; 3 Hospital Do Servidor Publico Municipal De São Paulo, Brazil; 4 Universidade Faderal De Pernambuco, Brazil; 5 University of Santiago De Chile (USACH), Chile; 6 Brazilian Society, Brazil; 7 Unifesp, Ufpr, Ufpe, UFMG, UFMT, Ufpa, Ufal

## Background

The majority of children with asthma develop symptoms such as wheezing in the first years of life. Low birth weight is considered as one of the risk factors for wheezing. The aim of this study was to describe the prevalence of wheezing during the first year of life in low birth weight infants, using the International Study of Wheezing in Infants (EISL).

## Methods

Parents of infants aged between 12 and 15 months, residents in six Brazilian cities: Belém, Belo Horizonte, Cuiaba, Curitiba, Recife and São Paulo, participated in interviews between 2005 and 2010 answered the questionnaire EISL. The infants were divided according to birth weight: normal weight (NW= 2,500g or more) or low birthweight (LW = less than 2,500g). Infants were also divided according to the frequency of wheezing episodes: recurrent wheezing (RWh, three or more episodes) and severe recurrent wheezing (SRWh, six or more episodes or were hospitalized for wheezing).

## Results

12,582 valid questionnaires were considered, and 11,411 (90.7%) infants were born with NW and 1,171 (9.3%) with LW. For the RWh, was 2,488 (21.9%) infants in the NW group and 331 (28.3%) in LW group (p <0.001). Presented SRWh, 1,491 infants (13.1%) of the NW group and 248 (21.2%) of LW (p <0.001) group.

## Conclusions

The prevalence of wheezing, especially in low birth weight infants is very high. Actions against smoking and complete pre-natal care would decrease the chances of the birth of low weight children.

